# Prevalence, knowledge and attitudes towards using sports supplements among young athletes

**DOI:** 10.1186/s12970-019-0294-7

**Published:** 2019-07-04

**Authors:** Pavle Jovanov, Višnja Đorđić, Borislav Obradović, Otto Barak, Lato Pezo, Aleksandar Marić, Marijana Sakač

**Affiliations:** 1 Institute of Food Technology in Novi Sad, University of Novi Sad, Bulevar cara Lazara 1, Novi Sad, 21000 Serbia; 2 Faculty of Sport and Physical Education, University of Novi Sad, Lovćenska 16, Novi Sad, 21000 Serbia; 3 Faculty of Medicine, University of Novi Sad, Hajduk Veljkova 3, Novi Sad, 21000 Serbia; 4 Institute of General and Physical Chemistry, University of Belgrade, Studentski trg 12-16, Beograd, 11000 Serbia

**Keywords:** Survey analysis, Correspondence analysis, Test of knowledge, Ethics in sports

## Abstract

**Background:**

The aim of this international study was to investigate the prevalence of the use of sports supplements among young athletes, as well as their knowledge and attitudes towards sports supplementation.

**Methods:**

Organized survey study testing the level of knowledge, attitudes, beliefs and practices concerning the use of sports supplements was administered to 348 athletes, 15–18 year olds from 4 countries competing in 18 sports at the international level.

**Results:**

The prevalence rate of the intake of sports supplements was 82.2%, with the protein supplements being predominant (54.5%). Coaches were identified as the primary source of information regarding supplementation (41.4%). The enhancement of athletic performance (35.4%) was the major motivation for the supplements intake. The majority of athletes (72.1%) were aware of associated health risks. The young athletes possess varying levels of knowledge regarding their own supplementation. The obtained data about the level of knowledge were statistically analyzed using the correspondence analysis. Less than 40% of athletes had the knowledge about the proper and intended use of protein, creatine, amino acids, beta alanine and glutamine, while they had greater understanding about vitamins and minerals, sports drinks and caffeine. The athletes in developed countries had greater access and utilization of professional resources such as dieticians. Young athletes are still unfamiliar with WADA regulations (55.5%), and the misuse of sports supplements represents an ethical dilemma for some.

**Conclusion:**

These findings indicate the necessity of a comprehensive education of all team members about sports supplements and careful supervision of the athletic development of young athletes.

**Electronic supplementary material:**

The online version of this article (10.1186/s12970-019-0294-7) contains supplementary material, which is available to authorized users.

## Background

Due to the development of novel training methodologies and media representation of professional sports, athletes from an early stage of adolescence have been raising the scale of competitive edge by employing different strategies. Sports nutrition represents the integration and application of scientifically-based nutrition and exercise physiology principles that support and enhance physical activity, athletic performance and recovery. Besides the implementation of sports nutrition and training strategies, athletes seek for some ergogenic aid, an external influence, which may just be the key impetus for victory. Dietary supplements are considered nutritional ergogenic aids, and the ones intended for the improvement of an athletic performance and faster recovery are known as sports supplements [1, 2].

Increased energy requirements are not regularly met in young athletes, especially during competition season; therefore, most of them are unable to make adequate nutritional choices for growth and development as well as for optimized athletic performance and rely on additional nutritional intake taken from sports supplements [1, 3–5].

The prevalence of sports supplements has rapidly increased over the last decade and the rate of new products availability on the market cannot be followed by the appropriate scientifically-based studies about their safety, quality and effectiveness [6–8]. Moreover, the increasing social acceptance of consumption of sports supplements may give some explanation of this phenomenon [8]. With the raising consumption of sports supplements there is also a need for more extensive education about these products [9]. Unfortunately, athletes rarely seek information from educated sources such as registered dietitians. Also, continuous educational programs on this subject are not available in every country, especially in the developing ones. This leaves athletes susceptible to misinformation which may lead to health problems and poor athletic performance [10]. The use of dietary supplements is also a risk factor for illicit substance use and may cause so-called inadvertent doping due to the contamination of their ingredients [11]. Another aspect worth considering is their effectiveness which is controversial [11, 12].

There are only a few studies published each year targeting the dietary supplementation in adolescents leading to insufficient resources and subsequent misjudgment of emerging trends in this field. Considering the participation of young athletes in the major sports events, it is important to know the patterns of sports supplements use among them in order to develop education programs towards avoiding unnecessary and indiscriminate supplements use [12].

Although many studies investigated the athletes self-reported level of knowledge about sports supplementation, in this study a different approach was used, i.e. testing of young athletes’ knowledge about the use and purpose of sports supplements according to the prevailing facts about sports supplementation [13].

Hence, the objectives of this study were: (a) to determine the prevalence of sports supplements, (b) to determine source of information regarding supplementation, (c) to assess beliefs and attitudes towards the use of sports supplements, (d) to estimate the level of knowledge with specifically defined survey questions and the reasons for taking supplements, (e) to identify trends or differences between categories of supplement users, and (f) to obtain an insight into young athletes’ ethical dilemma about the misuse of sports supplements.

## Methods

### Survey development and statistical analysis

This study was conducted in a period between March and November 2018. In this perspective study a design survey was used (provided as Additional file 1). Before each data collection, the study was announced a few days earlier in schools, sports clubs or international competitions. Coaches, teachers or parents of potential participants were contacted and introduced to the study in order to recruit athletes for the survey. The inclusion criteria were: the age between 15 and 18 and international competition level.

Three hundred and forty-eight athletes met the criteria and were surveyed, among which male and female participants were equally distributed. Also, the age distribution was balanced with half of the athletes of 15–16 years of age and the other half of 17–18 years of age. This international study included participation of young athletes from 4 countries: Serbia (39.4%), Germany (23.0%), Japan (20.1%) and Croatia (17.5%), all representing their countries at international competitions in 18 sports: kayak (27.9%), rowing (12.6%), canoeing (11.5%), basketball (8.6%), volleyball (8.6%), swimming (8.0%), athletics (4.0%), boxing (2.3%), soccer (2.3%), tennis (2.0%), karate (2.0%), handball (2.0%), water polo (1.4%), dance (1.4%), golf (1.4%), weightlifting (1.4%), archery (1.4%), and fencing (1.2%).

The survey consisted of 20 questions, divided into four main parts. The first part collected demographic and personal information on the study participants: age, sex, country, and the type of sport they are competing in. The second part obtained information regarding the usage, importance, source of information, safety and procurement of sports supplements. The third part tested the athlete’s knowledge about the proper use (timing, dosage and reason for use) of sports supplements. The last part investigated athletes’ beliefs and attitudes towards the use of sport supplements and possible Anti-Doping rules violations.

Athletes voluntarily completed the written survey on different occasions and places such as: international competitions, high schools or on individual basis at different sport clubs. The survey was previously reviewed by various certified coaches in different sports, physicians, university professors and researches specialized in food science and sport psychology.

The reliability analysis of the survey items revealed that all variables measured were reliable with reliability values of all the latent variables extracted above 0.7 (for Cronbach’s Alpha). The Composite Reliability (CR), which represents the overall reliability of a multi-dimensional construct reached values above 0.9, which is attributed as particularly significant. Data were normally distributed and negatively skewed with relatively flat peak. Average Variance Extracted (AVE) was estimated, and the significant values above 0.5 were obtained, meaning that the latent variables were bringing significant variation in the face of random measurement error.

All three conditions of convergent validity were satisfactorily met, i.e. regression weights/factor loadings were equal to or greater than 0.5, whereas squared multiple correlations (SMC) were equal to or greater than 0.7, while AVE values were equal to or greater than 0.5. All aforementioned conditions confirmed the convergent validity of the constructs. In order to test whether two constructs differ from each other, discriminant validity of the constructs was also checked and confirmed by showing that AVE was greater than SMC for each variable.

All surveyed athletes were previously informed about the study objectives and had a chance to clarify any possible misunderstanding of the survey questions with the team conducting the study. While filling out the survey a representative of the team conducting the study was present at the site.

This study was approved by the Ethics Committee of the Faculty of Medicine, University of Novi Sad, and all procedures were conducted in accordance with the Declaration of Helsinki.

Data were processed using Microsoft Excel (Microsoft Corporation, Redmond, Washington, USA) and analyzed using the statistical software Statistica 12 (Dell Software, Round Rock, Texas, USA). Descriptive data were calculated as frequencies. Data were evaluated by sex and age using chi-square (χ^2^) analyses. Significance was determined at *p* < 0.05. For the statistical analysis, two age categories were used: athletes 15–16 year olds (15-16Y) and athletes 17–18 year olds (17-18Y). The collected data about the proper use of sports supplements among different demographics were analyzed using the correspondence analysis. This analysis is a useful statistical technique for analyzing data collected in sport surveys by simple graphical presentation with a set of points with respect to two coordinate axes [14]. Symmetric normalization model [15–17] was suitable for exploring relationships between items of two nominal variables.

## Results

### Prevalence of the use of sports supplements

The survey showed that 82.2% of athletes were using sports supplements among which 60.6% were male athletes. The analysis revealed that 47.7% of athletes were 15–16 year olds (*p* = 0.038) and 52.3% were 17–18 year olds (*p* = 0.032). Furthermore, male athletes were more prone to the use of sports supplements in both age categories (56.8 and 64.0% in 15-16Y, (*p* = 0.029) and 17-18Y, (*p* = 0.021), respectively).

**Fig. 1:**
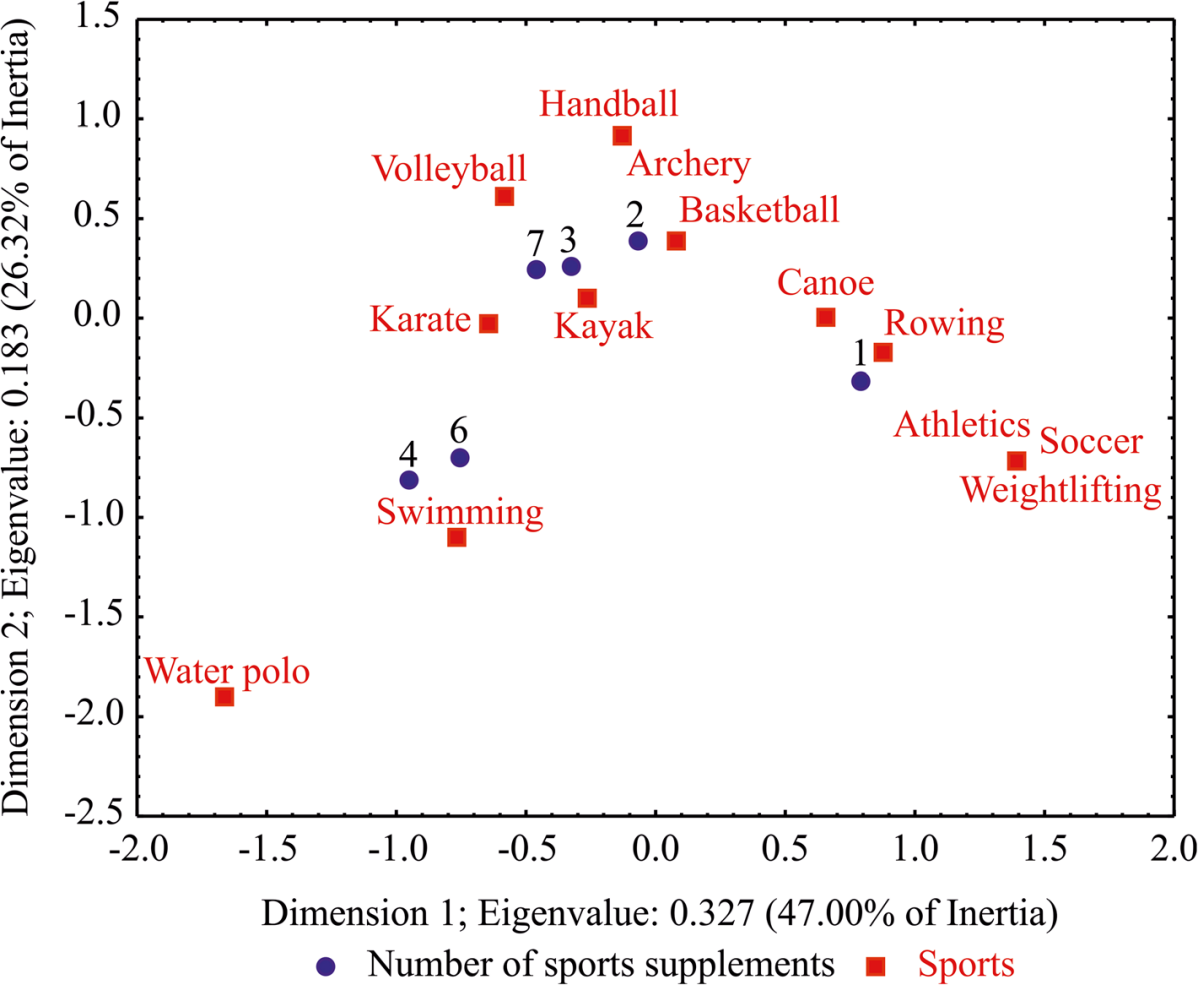
Correspondence analysis – the first dimension distinguishes between different sports, while the second dimension presents the differences in a number of consumed sports supplements among different sports

The study revealed that 82.2% of athletes used 1–2 different supplements at the same time, 62.1% 2–3, and 35.9% 3–4, while 14.7% of athletes used 4 and more. Biplot in Fig. 1 shows the projection of the correspondence analysis (total inertia of 0.6955, χ^2^ of 64.682, *p* = 0.007) of number of supplements taken by different sports, among which kayak, swimming and karate were identified as the one with the highest number.

**Fig. 2:**
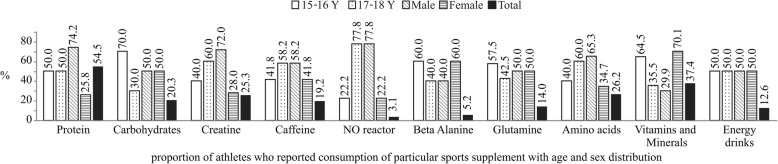
Prevalence of sports supplements – percentage of athletes using a specific sports supplement marked as total bars. The age and sex distribution within specific supplement is represented by corresponding bars marked as male/female (100%) and 15-16Y/17-18Y (100%)

Figure 2 shows that whey protein usage by 54.5% of athletes can be observed, together with the prevalence of ten other sports supplements. The males use more (*p* = 0.030) whey protein, creatine, amino acids, caffeine and NO reactor compared to females who take more vitamins and mineral complexes, while there is an almost equal use of energy drinks, glutamine and carbohydrates between sexes. Between the age categories the use of protein supplements and consumption of energy drinks were equally distributed; younger athletes tend to use more carbohydrates, beta alanine, glutamine, vitamins and mineral complexes versus 17-18Y athletes who take more creatine, caffeine, NO reactor and amino acids.

### Reasons for the use, attitudes, supplement source and source of information regarding sports supplements

**Fig. 3:**
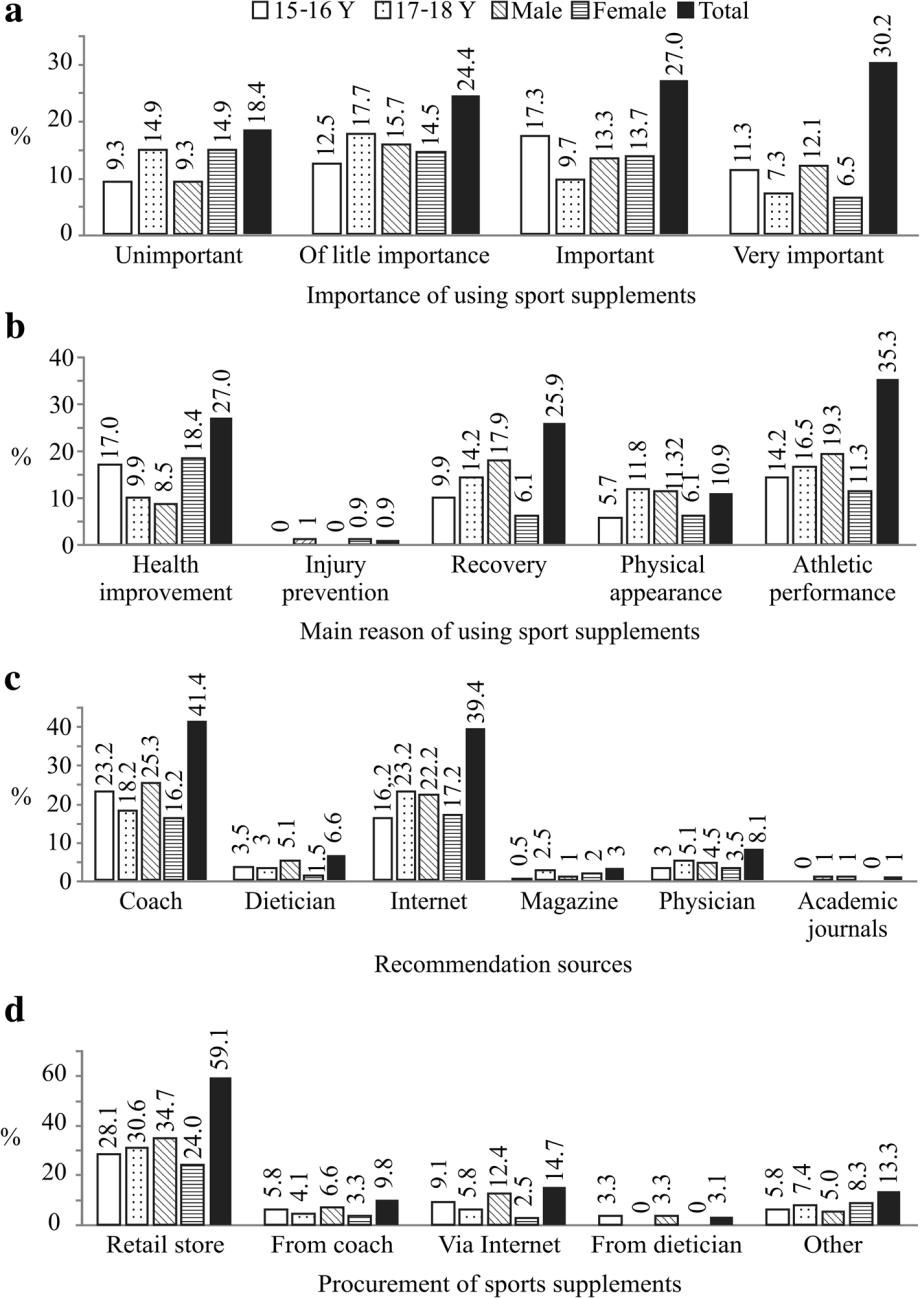
**a** Attitudes, **b** beliefs, **c** impact on supplementation and **d** procurement of sports supplements

When asked how important good nutrition and proper supplementation is for enhancement of athletic performance 30.2% of athletes thought that it is very important, while 18.4% though it is unimportant (Fig. 3a).

The main reasons the athletes gave for taking supplements are presented in Fig. 3b with the improvement of their athletic performance (35.3%) being the predominant one. Female athletes were significantly (*p* = 0.047) more likely to take supplements “for their health” while males use it for boosting of athletic performance.

The attitudes of the athletes who do not take supplements were expressed through the following statements: I don’t need them (48.4%); I don’t know enough about them (21.0%); they are unhealthy (14.5%); they are expensive (8.1%); using supplements is like cheating (4.8%); they are not allowed (1.6%), I fear of a positive doping test (1.6%).

Considering the “unclear picture” regarding the health safety and quality of sports supplements and their impact on athlete’s health, 72.1% of athletes were aware of a certain health risk, 14.9% thought they are risky and 12.9% of athletes consider them safe.

Supplement safety information were gathered from the coach (38.2%), dietician or medical professional (33.3%) or the declaration on the product (20.1%). The athletes rely less on their own research about the health impact of the sports supplements (8.3%).

The majority of athletes, mostly males and 15-16Y reported that they obtained information regarding sports supplements from their coach (41.4%); likewise 17-18Y athletes rely on the Internet as shown in Fig. 3c.

Athletes mostly procure sports supplements in specialized retail stores (59.1%) as shown in Fig. 3d. There was no significant statistical difference (*p* > 0.05) between age categories concerning the answers to the question where they buy sports supplements. However, male athletes tended to use more online shopping in acquiring sports supplements than females.

### Knowledge about proper and intended use of the sports supplements

**Fig. 4:**
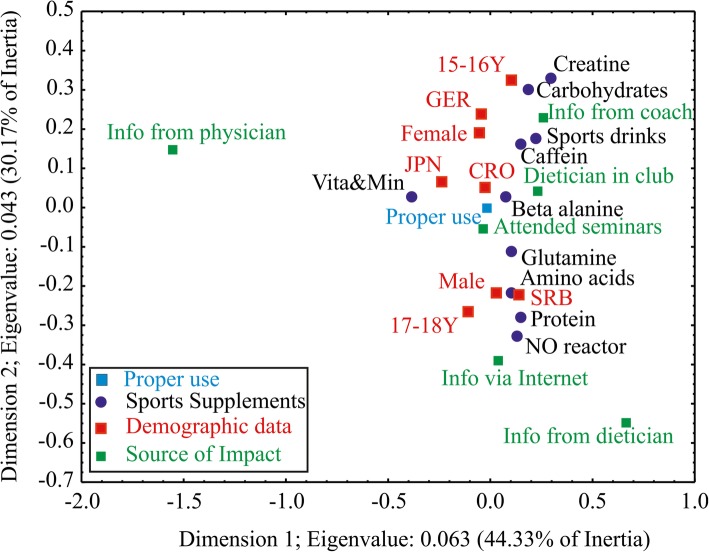
Correspondence analysis – the first dimension explains differentiation among proper use of sports supplements regarding the sources of impact while the second dimensions explains the differences in demographics data of athletes regarding different sports supplements

One of the main goals of this international study was to assess the young athletes’ understanding of the proper and intended use of the sports supplements. The level of knowledge was assessed by conducting an enquiry about the proper timing (before, during or after training), right serving amounts and the main reason for their use. Graphical presentation of correspondence analysis is presented in Fig. 4. A significant correspondence (*p* = 0.008) was found between the considered categories, representing the total inertia of 0.141 and χ^2^ value of 214.88. The first two dimensions account for 74.5% of the total inertia. Substantial differentiation between the proper use and sources of information among different demographics can be observed.

Unfortunately, young athletes lacked proper knowledge about the use of creatine (11.1% of athletes responded correctly), beta alanine (20.0%), amino acids (20.0%), NO reactor (22.2%), glutamine (37.5%), protein (38.5%), and carbohydrates (48.3%). Yet they seem to have more knowledge about sports drinks (50%), caffeine (61.8%) and vitamins and mineral complexes (71.0%). Previous attendance at educational seminars influenced the right answers about the use of creatine (75% of athletes previously attended seminars), amino acids (66.7%), carbohydrates (57.1%), proteins (55.0%), vitamins and minerals (52.6%), caffeine (50.0), NO reactor (50%), glutamine (46.7%), sports drinks (44.4%) and beta alanine (33.3%).

Athletes 17-18Y showed better knowledge than 15-16Y (*r* = 0.968; *p* < 0.001), as well as female athletes compared to males (*r* = 0.953; *p* < 0.001). Athletes from all 4 countries were among the ones who knew the proper use of supplements: Serbia (*r* = 0.9013, *p* = 0.003), Germany (*r* = 0.9302, *p* < 0.001), Japan (*r* = 0.954; *p* < 0.001) and Croatia (*r* = 0.979; *p* < 0.001). Younger athletes (15-16Y) had better understanding of the proper use of creatine (87.5%), carbohydrates (67.9%), sports drinks (66.7%), beta alanine (66.7%), and caffeine (55.9%), while the older athletes (17-18Y) gave the right answers when it comes to the proper use of glutamine (60.0%), vitamins and minerals (60.5%), proteins (70.0%) and amino acids (86.7%). Male athletes were better informed on the right use of NO reactor (100.0%), creatine (75.0%), amino acids (66.7%), protein (60.0%) and glutamine (53.3%), while the female participants were more educated in the proper use of caffeine supplements (73.5%), carbohydrates (71.4%), beta alanine (66.7%), vitamins and minerals (56.6%) and sports drinks (55.6%).

The coach was the only source of information regarding the proper use of creatine. The coach was also the sole source of information for 83.1% of athletes about proper carbohydrates use, amino acids (73.3%), caffeine (73.3%), sports drinks (72.2%), glutamine (53.3%), NO reactor (50.0%), protein (40.0%), beta alanine (33.3%), and vitamins and mineral complexes (32.9%). The 15-16Y athletes took supplements properly according to the coach’s advice (*r* = 0.912; *p* = 0.003). The older athletes (17-18Y) gathered information from the Internet (*r* = 0.942; *p* < 0.001) and have attended the seminars about that topic (*r* = 0.963; *p* < 0.001). Athletes in Serbia had a better understanding of the proper use of proteins (50.0% of right answers), while athletes in Japan were better educated on the use of vitamins and minerals (32.9%). Furthermore, athletes from Germany had a better understanding of carbohydrates (35.7%) and creatine (50.0%). Croatian athletes showed good recognition of sports drinks (50.0%).

The physician was the source of information for 36.8% of athletes about vitamins and mineral complexes, while others used the Internet. Athletes who used sports supplements properly attended more seminars about sports supplementation than others (*r* = 0.967; *p* < 0.001). Male athletes gathered the information on how to use sports supplements using the Internet (*r* = 0.951; *p* < 0.001) and attending seminars (*r* = 0.961; *p* < 0.001), while female athletes were mostly advised by their coaches (*r* = 0.892; *p* = 0.007). Also, athletes from Serbia used the Internet as a source of information more than athletes from other countries, who knew the proper way of using sports supplements (*r* = 0.971; *p* < 0.001).

Only 27.9% of all surveyed athletes had the opportunity to work with dieticians in their sports clubs, but only 20.0% of those who answered correctly about the proper use of sports drinks and proteins and 3.6% about carbohydrates used that opportunity. Mostly, athletes from Germany had a dietician in their clubs (*r* = 0.778; *p* = 0.006). Athletes in Germany and Japan who answered correctly about the use of supplements attended more seminars (*r* = 0.927; *p* = 0.004 and *r* = 0.923; *p* = 0.004, respectively) and utilized more advice from dieticians, rather than athletes from other countries (*r* = 0.824; *p* = 0.003 and *r* = 0.882; *p* = 0.003, respectively).

### Risks of doping and ethical dilemma

Awareness and caution about possible risks of doping is the key for proper supplement use, following regulations of the World Anti-Doping Agency (WADA). This study revealed that only 55.5% of athletes had the access and are familiar with these regulations. When asked if they would be willing to use prohibited substance to enhance their athletic performance if they knew that they would not be tested by WADA, 11.8% of athletes gave a positive answer.

## Discussion

This study dealt with the prevalence, tested level of knowledge and ethical dilemmas about the consumption of sports supplement among young elite athletes competing at the international level in 18 different sports from 4 countries.

### Prevalence and beliefs

The percentage of athletes using sport supplements in this study (82.2%) is in an agreement with the studies who pointed out high supplement consumption among young athletes [5, 10, 18, 19]. Furthermore, similar prevalence can be observed in 87.5% of Australian athletes [8], 77.0% of Singaporean athletes [13], and 71.2% of USA adolescents [20], while more than half of the British athletes (62.0%) and 45.0% of Iranian athletes [6] take some type of a sports supplement [21]. On the contrary, Nabuco et al. [12] reported that only 47.3% of Brazilian athletes use sport supplements. Scofield and Unruh [22] reported that only 22.3% of young USA athletes consume supplements. The overall prevalence rate of sports supplements differs between studies, and a possible explanation can be found in variable sample size, age category and different level of competition among athletes. The results in this study clearly show that the prevalence of sports supplements increases with age and that supplementation is more preferable choice of male athletes.

High percentage of athletes consuming more than four supplements (14.7%), which was found in this study, can be compared with 15.1% reported by Nabuco et al. [12], raising the awareness about possible health implications among youth. Dascombe et al. [8] found that kayakers and swimmers use considerably higher number of supplements compared to other investigated sports, which is in agreement with this study exploring the possibility that athletes in individual sports rely more on supplementation than athletes in team sports. The necessity of using different energy systems during sporting events can result in an increased number of sports supplements in these sports.

Prevalence of whey protein in this study deviates from the one of 21.7% reported by Froiland et al. [10]. However, the consumption of whey protein increased over the last two decades [23], first to 30% in 2006 [19] than 53.5% in 2014 [12] and finally 54.5% in this study. In reaching new world records, current intensive training regimes demand higher protein intake for greater metabolic adaptation, better remodelling and faster tissue repair. Balanced meal plans do not usually meet these requirements, while the additional protein intake satisfies these needs and provides a comfortable choice for young athletes who do not spend time preparing their meals [1]. Creatine is one of the most popular sports supplement these days and it is consumed by 25–40% of young athletes [7, 10, 12, 19]. A broad range of creatine use can be attributed to its greater representation in sports where strength and speed are imperious [24]. The use of vitamins and mineral complexes was reported by 37.4% of athletes, which is similar to 45.0% of Australian athletes [8] and 45–47% of UK athletes [7, 21]. These results differ from the prevalence higher than 80% found in other studies [19]. The possible mismatch of the total share can be attributed to the inclining use of other sports supplements compared to seemingly same amounts of vitamins and mineral complexes used over last decade.

The majority of young athletes (57.2%) believe that supplementation is important for sport success which is opposite to 78.4% athletes in study of Petróczi et al. [7] who did not attribute the importance of supplementation. However, the change of the attitude can be attributed to increasing media influence on the sports supplements market.

One of the main reasons behind the use of supplements is enhancement of athletic performance. Results found in this study corroborate the results of other studies which elaborated the same reasoning [4, 6, 8, 12]. Health concern is also a strong motivation, but only 27.5% of athletes think so, which is in agreement with the study of Nieper [21], and in contrast to high percentage of athletes in other studies [5, 18, 19] who found that health concern was the main reason behind taking sports supplements. Although young German athletes were surveyed in the study of the Braun et al. [19] and in this study, the ones in this study were more focused on boosting their athletic performance.

There is a high percentage of athletes who are not supplement users and the most frequent reason they stated was that they had no need for supplementation. Similar reasoning was found in other studies [4, 12, 21]. Lack of knowledge was another strong reason for avoiding supplements, and this study confirms that the greater the knowledge about supplementation is, there is more willingness in an athlete to use supplements [21].

Young athletes agree on one thing; some sports supplements carry certain health risks. 72.1% of athletes in this study shared that belief with 83% of UK young athletes [21], and the decision on whether the supplement is safe is based mostly on the advice from the coach. In line with other studies [4, 7, 11, 12, 19, 21, 22, 25–28] the coach is a primary source of information about the supplements, which is somehow expected since the athletes in early stages of their semi-professional or professional sport career are very emotionally attached to their coaches and spend a lot of time with them. However, these findings emphasize the need for the enforcement of education programs for coaches about sports supplements.

### Knowledge about the proper use of supplements

Based on the correct responses on proper and intended use of sports supplements, the study participants exhibited a relatively low level of knowledge in domains of sports supplementation. The young athletes had issues related to understanding of roles and intended benefits of different supplements. The results of this study corroborate the study of Tawlik et al. [4] who encountered the same misconception about the role of proteins as an energy drive substance for physical activity and not for muscle growth and repair. The lack of congruence between intended use and perceived knowledge was also observed by Petróczi et al. [7]. Consequently, ongoing education about the roles of nutrients is advised. Other studies tried to assess the knowledge by investigating self-perceived knowledge by athletes. In one by Dascombe et al. [8] 36.0% of athletes were still largely uneducated with regards to their sports supplement routine and in another by Slater et al. [13] more than 60% had little or limited knowledge about the subject. As it can be noticed the coach and the Internet were the main sources of information for the athletes who properly used supplements. The relationship between the coach and the athlete was already addressed, and the Internet has become the main source of information, so it is not surprising that young athletes also utilize that resource. It is worth mentioning that the education of coaches on this subject is of great importance considering that many coaches do not have enough knowledge to give appropriate supplements recommendations [29, 30]. Compared to 75.0% of athletes in the study by Nieper [21] who had access to sports dieticians, only 27.9% of them in this study had the same opportunity. This stresses the underrepresentation of specialized staff in sports clubs, especially in the developing countries. One of the objective reasons may lie in the fact that many sport clubs do not have enough financial resources to have qualified professionals such as dieticians at their disposal. However, both studies confirm a low utilization of their services, and a possible explanation may lie in the fact that the athletes are not familiar enough with the spectrum of the services that dieticians provide.

### Doping and ethics

Without a proper guidance when taking dietary supplements there is a great risk of a positive doping and adverse effects on athletes’ health. Knowledge and implementation of WADA regulations is nowadays mandatory for any athlete competing at the international level. The percentage of athletes familiar with these regulations implicates the need for further implementation of these regulations in all educational programs. However, the authors did not find any study concerning ethical questions about possible doping. This study provides one of the first results about ethical concerns in youth sports, showing that 11.8% of athletes would use banned substances for the advancement in sports. Many reasons could be found behind that rationale, but the importance of ongoing education about possible health implication of supplements, banned or allowed ones, is definitely one of the main strategies in changing that perspective.

### Study limitations

Possible study limitation can be found in an uneven number of participants from each country and the distribution of athletes between sports. Hence, the comparisons were made mostly by sex and age; however, the results obtained in this study may serve to obtain valuable guidelines towards future global trends about sports supplementation. Comparisons made between sports were excluded from this study due to the excessive extent that this manuscript would have given the specifics of each sport, and the sociological differences between them that influence the choice and manner of supplements intake. However, the results about the number of supplements used in each sport were included to underline valuable trends in supplements consumption among different sports.

## Conclusion

In conclusion, the results of this survey study indicate that supplementation is widespread among young athletes (82.2%) and not restricted to specific demographics or sport. The athletic performance is the main motivation, however the level of knowledge young athletes have about the proper and intended use of sports supplements reveals the knowledge gap and the necessity of enforcing ongoing education about sports supplementation.

### Practical applications

- The protein supplements are widespread among young athletes.
- The coach is the main source of information about supplementation practices
- The enhancement of athletic performance is the main reason of using sports supplements by young athletes.
- The level of knowledge about the proper and intended use of sports supplements is inadequate.
- Ongoing education about sports supplements by all athletes and coaches is necessary for maximizing athletic performance and minimizing the risk of positive doping test.
- Insufficient knowledge causes ethical dilemma about the misuse of sports supplements.

## Additional file

Additional file 1: The survey. (XLSX 28 kb)

## Data Availability

The datasets used and/or analysed during the current study are available from the corresponding author on reasonable request.
